# Time-Dependent Material Properties of Shotcrete: Experimental and Numerical Study

**DOI:** 10.3390/ma10091067

**Published:** 2017-09-11

**Authors:** Matthias Neuner, Tobias Cordes, Martin Drexel, Günter Hofstetter

**Affiliations:** 1 Unit for Strength of Materials and Structural Analysis, Institute of Basic Sciences in Engineering Sciences, University of Innsbruck, Technikerstr 13, A-6020 Innsbruck, Austria; Martin.Drexel@uibk.ac.at (M.D.); Guenter.Hofstetter@uibk.ac.at (G.H.); 2 Brenner Basetunnel BBT SE, A-6020 Innsbruck, Austria; Tobias.Cordes@bbt-se.com

**Keywords:** shotcrete, sprayed concrete, material tests, constitutive model, creep, shrinkage, damage model, plasticity model

## Abstract

A new experimental program, focusing on the evolution of the Young’s modulus, uniaxial compressive strength, shrinkage and creep of shotcrete is presented. The laboratory tests are, starting at very young ages of the material, conducted on two different types of specimens sampled at the site of the Brenner Basetunnel. The experimental results are evaluated and compared to other experiments from the literature. In addition, three advanced constitutive models for shotcrete, i.e., the model by Meschke, the model by Schädlich and Schweiger, and the model by Neuner et al., are validated on the basis of the test data, and the capabilities of the models to represent the observed shotcrete behavior are assessed. Hence, the gap between the the outdated experimental data on shotcrete available in the literature on the one hand and the nowadays available advanced shotcrete models, on the other hand, is closed.

## 1. Introduction

The proper representation of the constitutive behavior of shotcrete is an important prerequisite for realistic numerical simulations of deep tunnels driven by the New Austrian Tunneling Method. In the past 30 years, several approaches for describing the complex, time-dependent material behavior of shotcrete have been presented in the literature. Besides the commonly employed approach in practice, based on linear elasticity with a hypothetical Young’s modulus for shotcrete, to consider time-dependent material behavior and loading in an approximate manner [1], two of the most widely used shotcrete models are the viscoplastic model by Meschke [2] and the viscoelastic-plastic model by Schädlich and Schweiger [3]. Recently, a new damage plasticity model for shotcrete and a comparison of the latter with the models by Meschke and by Schädlich and Schweiger were published by Neuner et al. [4] and the performance of the aforementioned models was compared on the basis of a 2D benchmark example of deep tunnel advance in [5].

However, most of the papers on constitutive models for shotcrete rely on rather old experimental data for validation and evaluation of the proposed models. These quite old experimental programs comprise (i) the evolution of the Young’s modulus and the uniaxial compressive strength up to the age of 28 days and short-term uniaxial and triaxial compression tests on shotcrete specimens of different age by Sezaki et al. [6], (ii) the evolution of the uniaxial compressive strength up to the age of 28 days and stress–strain relations from uniaxial compression tests and the evolution of the total strain in creep and shrinkage tests by Aldrian [7] and Golser et al. [8], (iii) the evolution of temperature due to hydration, of the Young’s modulus and of the uniaxial compressive strength up to the age of 7 days as well as the evolution of the total strain in shrinkage and creep tests, reported by Huber [9], (iv) the evolution of the Young’s modulus and uniaxial compressive strength and the results of relaxation and shrinkage tests up to the age of 7 days by Fischnaller [10] and (v) the evolution of stiffness and strength, short-term uniaxial compression tests and experiments for determining the total strain in shrinkage and creep tests by Müller [11].

More recently, experimental studies on the bond strength between shotcrete and sandblasted concrete [12] and on the bond strength between shotcrete and hard rock [13] were published. In the former, additionally the evolution of the uniaxial compressive strength up to the age of 28 days and the drying shrinkage behavior up to the age of 112 days is described, whereas in the latter only the evolution of the uniaxial compressive strength up to the age of 72 h is reported. In addition, a detailed summary of experimental results on shotcrete available in the literature and a discussion can be found in [14].

However, the available experimental data on the time-dependent material behavior of shotcrete is limited and many of the reported experimental data sets are outdated by today, since nowadays different shotcrete compositions accompanied by improved technology are used. These shortcomings motivated the design of a new a experimental program.

The paper is structured as follows: in the next two sections, the experimental program for determining material data of shotcrete and the test results are described. In the subsequent sections, the shotcrete models by Meschke [2], Schädlich and Schweiger [3] and Neuner et al. [4] are reviewed briefly, the calibration of the shotcrete models on the basis of the presented experimental data is described and their ability to reproduce the experimental data is evaluated.

## 2. Experimental Program

The experimental program on shotcrete comprises the evolution of (i) the Young’s modulus and (ii) uniaxial compressive strength and the time-dependent development of the total strain in (iii) shrinkage tests and (iv) creep tests, both on sealed specimens.

The employed shotcrete composition, designated as *SpC25/30/(56)/ÜK3/J2/XC4/XF3/GK8* according to the Austrian guideline for shotcrete in [15], is listed in Table 1. *SpC25/30/(56)* characterizes the shotcrete strength class, *ÜK3* the monitoring class, *J2* the early age strength class according to [16], *XC4* and *XF3* the exposure classes, and *GK8* a maximum aggregate size of 8 mm. The composition investigated in the present study is representative for commonly employed modern shotcrete compositions used within the New Austrian Tunneling Method.

All specimens were sampled at the site of the Brenner Basetunnel during the securing measures of tunnel advance with a shotcrete shell. The specimens were taken from three different locations of the tunnel face in order to capture the variability of shotcrete properties due to the adjustment of the amount of the added accelerator at the tunnel site according to current requirements and ambient conditions.

**Figure 1:**
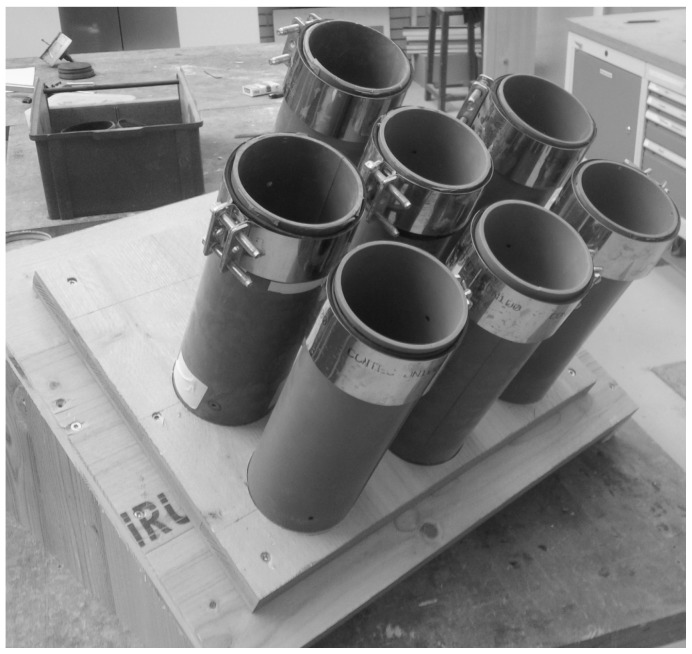
Molds for sprayed specimens: slotted tubes, fixed with a clamp on the upper side and mounted on a wooden rig.

The tests were conducted on two different types of specimens: (i) drill cores sampled from spray boxes, which is the commonly employed type of specimen for determining material properties of shotcrete, and (ii) cylindrical specimens directly sampled from tubular molds, denoted as *sprayed* specimens. For the latter longitudinally slotted PVC tubes, mounted on wooden rigs, were used as molds (Figure 1). They were closed in circumferential direction by metal pipe clamps and the operator sprayed the shotcrete directly into the PVC tubes. The clamps were removed at unmolding of the specimens. The sprayed specimens were used for tests on shotcrete younger than 24 h, since drill cores cannot be sampled for this early shotcrete age due to the low material strength. The quality of the sprayed specimens was evaluated by means of visible cavities at the surface. Faulty specimens due to shotcrete rebound and air pockets were sorted out. Suitable specimens were obtained if the spraying direction was nearly coaxial with the axis of the tubular molds. For the present shotcrete composition, sprayed specimens could be unmolded safely already 6 h after casting.

A basic requirement for experiments are smooth top and bottom surfaces of the specimens, which are parallel to each other. Since cutting was impossible at young shotcrete ages, compensation layers, consisting of a fast-curing epoxy resin, were applied onto the top surfaces of the specimens. No treatment of the bottom surfaces was necessary.

Both drill cores and sprayed specimens had a diameter of 100 mm. The length of specimens for determining the Young’s modulus and the uniaxial compressive strength was 200 mm, while specimens for shrinkage and creep tests had a length of 300 mm. The number of intact specimens for each type of test is provided in Table 2.

All specimens were sealed and stored in an air-conditioned chamber with an ambient temperature of (20 ± 1) °C until testing. The same constant ambient conditions were ensured during the long-term shrinkage and creep tests.

Experiments on the evolution of the uniaxial compressive strength were started at the shotcrete age of 6 h, while tests on the evolution of the Young’s modulus were started at the shotcrete age of 24 h. Both tests were performed up to the shotcrete age of 28 days. For shotcrete younger than 24 h, exclusively sprayed specimens were used. For tests on shotcrete older than 24 h, both types of specimens were used. Between the two specimen types, no significant differences of test results were detected and, thus, no distinction between them is made in the following. In contrast, Müller [11] observed differences in the determined material properties for different types of specimens.

**Figure 2:**
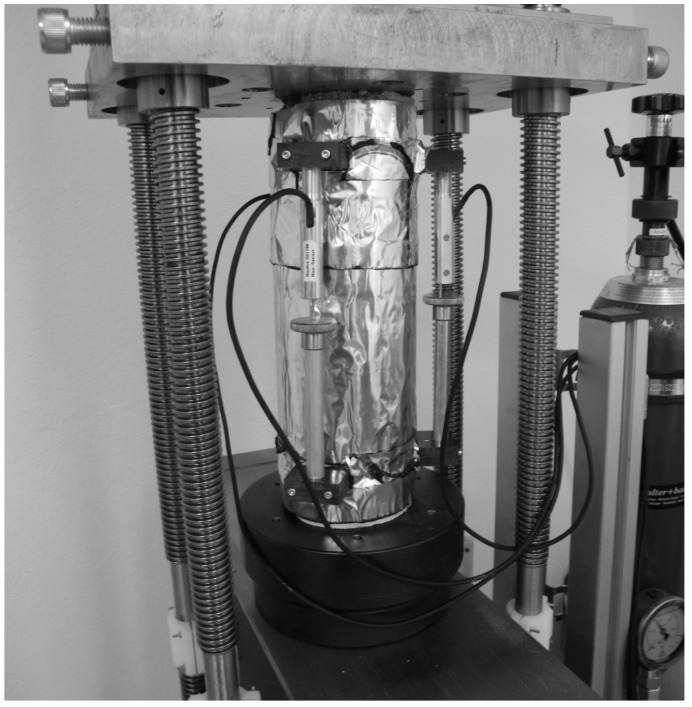
Sealed specimen in the creep test bench with displacement transducers fixed to the specimen.

Tests for determining shrinkage and creep of shotcrete were only performed on sealed, sprayed specimens. After unmolding, the specimens were sealed using a bitumen–aluminum foil in order to prevent drying. The creep tests were started at different shotcrete ages, i.e., 8 h, 24 h and 27 h, in order to investigate the influence of the material age at load application on the creep behavior. A shotcrete age of 27 h (instead of 24 h) had to be chosen for scheduling reasons. Creep tests were carried out with a hydraulic creep test bench (Figure 2). The load was applied within 2 s, and was held constant for 56 days. This is in contrast to many creep tests on shotcrete reported in the literature, which are characterized by a stepwise increase of the sustained stress in order to simulate the further loading of shotcrete due to tunnel advance. However, it is in accordance with the recommendation by Sercombe et al. [17], who criticized the commonly employed stepwise increase of the sustained load because it complicates the calibration of shotcrete models. The magnitude of the applied compressive stress was chosen such that linear viscoelastic shotcrete behavior was ensured. For each creep test, the shotcrete age at application of the sustained compressive stress and its magnitude are provided in Table 2. Each creep test was accompanied by one shrinkage test, which was started simultaneously. In the shrinkage and creep tests, the time-dependent length changes were measured by three inductive displacement transducers with a measurement length of 200 mm, fixed to the specimens at 0°, 120°, and 240° along the perimeter of a specimen. The reported strain of a specimen is computed from the mean value of the three measured length changes and, thus, it represents the total strain at the center of the specimen.

## 3. Experimental Results

**Figure 3:**
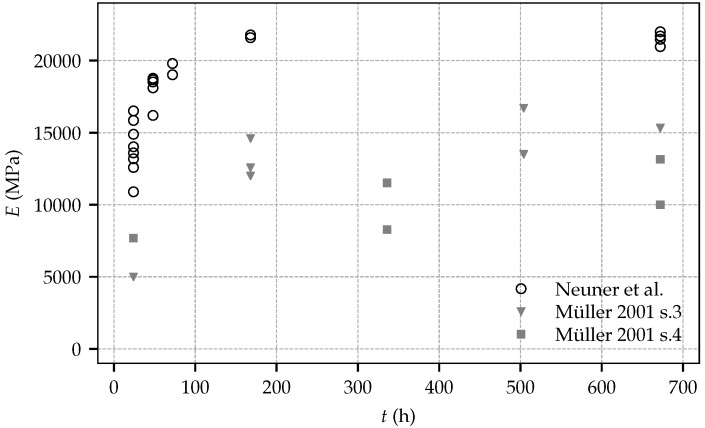
Evolution of the Young’s modulus: experimental results from the present experimental program and test data by Müller.

Figure 3 shows the measured Young’s modulus at shotcrete ages according to Table 2, and, in addition, for comparison, the respective experimental results on the evolution of the Young’s modulus presented by Müller [11], determined in two test series, designated as test series 3 and 4. The shotcrete composition investigated by Müller is characterized by a water content of 150 kg/m^3^, an aggregate content of 1768 kg/m^3^ (with a maximum aggregate size of 8mm) and a cement content (manufacturer designation *SBM W&P)* of 340 kg/m^3^. Comparing the results presented by Müller and those of the present study reveals the improved performance of modern shotcrete compositions: compared to the shotcrete tested by Müller, the specimens sampled during the current experimental program exhibit a higher Young’s modulus throughout the considered time period. Furthermore, in the present experimental program, the stiffness at the age of seven days is already equal to the one at the age of 28 days. From the experimental data mean values of the Young’s modulus at the ages of one day and 28 days are identified as $E^{\left(1\right)}$ = 13,943 MPa and $E^{\left(28\right)}$ = 21,537 MPa, respectively.

**Figure 4:**
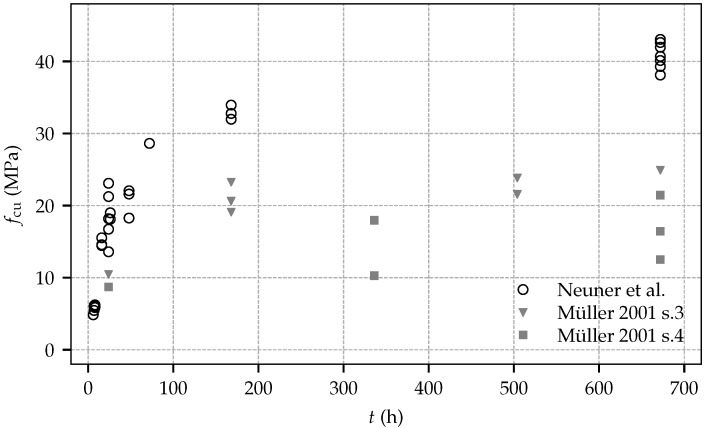
Evolution of the uniaxial compressive strength: experimental results from the present experimental program and test data by Müller.

Figure 4 contains the measured uniaxial compressive strength at shotcrete ages according to Table 2, and, for comparison, the respective experimental data from test series 3 and 4 by Müller [11]. Similar to the tests on the evolution of the Young’s modulus, the observed uniaxial compressive strength in the present experimental program is substantially higher compared to the one determined by Müller. Contrary to the evolution of material stiffness and in contrast to the experimental data on material strength by Müller, for the present experimental program, a considerable increase of strength can be observed between the age of seven days and 28 days. From the experimental data, mean values of the uniaxial compressive strength at the ages of one day and 28 days are identified as $f_{\mathrm{cu}}^{\left(1\right)}$= 18.57 MPa and $f_{\mathrm{cu}}^{\left(28\right)}$ = 40.84 MPa, respectively.

Comparing the material properties determined in the present experimental program and in the one presented by Müller reveals a noticeable higher stiffness and strength of the shotcrete employed in the present experimental program. These differences can be attributed to further developed shotcrete technology and accordingly improved shotcrete compositions.

**Figure 5:**
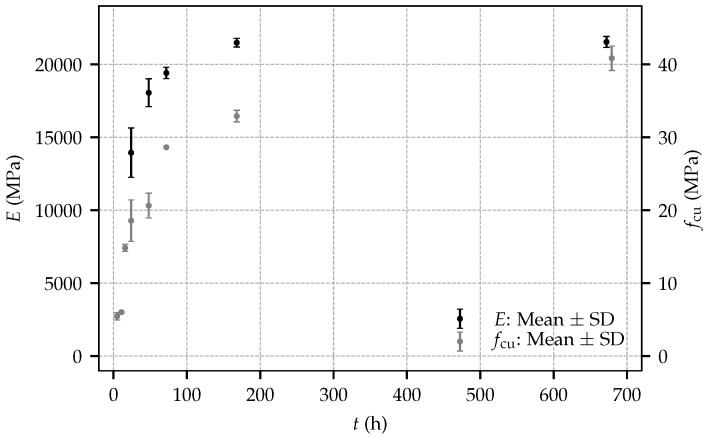
Mean values and standard deviations (SD) of the Young’s modulus and the uniaxial compressive strength for the present experimental program.

The mean values and the standard deviations (SD) of the Young’s modulus and the uniaxial compressive strength at different shotcrete ages are provided in Table 2, and are illustrated in Figure 5. For both material properties, the largest scatter is observed at the shotcrete age of 24 h. Remarkably, the determined values of the uniaxial compressive strength at even younger ages are in very good agreement with each other. Regarding the evolution of material stiffness, the standard deviation of the Young’s modulus is decreasing gradually up to the age of seven days.

**Figure 6:**
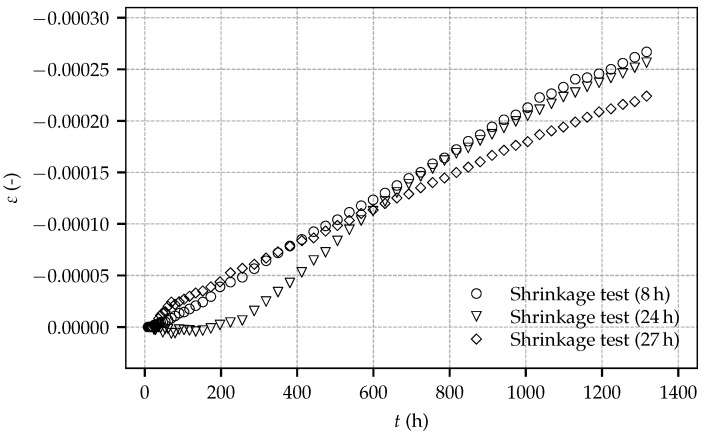
Evolution of the total strain determined on sealed, load-free specimens: experimental results for the tests on three specimens of the present experimental program, started at shotcrete ages of 8 h, 24 h and 27 h.

Figure 6 shows the evolution of the total strain determined in the shrinkage tests on sealed specimens. The total strain consists of the autogenous shrinkage strain and the thermally induced strain due to hydration. It follows from this figure that, for one specimen, an initial delay of the evolution of the identified total strain is observed, whereas, for the other two specimens, the expected evolution of the total strain right after the start of the experiments occurred. The origin of the initial delay remains unclear; however, the measured rate of the shrinkage strains is similar for the three specimens. Furthermore, the determined total strain is similar to the respective strain observed by Huber [9] in shrinkage tests on sealed prismatic specimens with dimensions of 100 mm × 100 mm × 400 mm.

**Figure 7:**
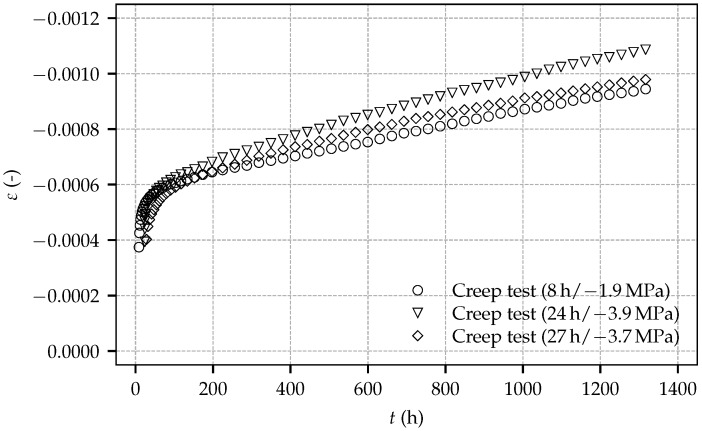
Evolution of the total strain determined on sealed, loaded specimens: experimental results for the tests on three specimens of the present experimental program, started at shotcrete ages of 8 h, 24 h and 27 h.

Figure 7 depicts the evolution of the total strain determined in the creep tests on sealed specimens loaded by sustained compressive stresses of 1.9 MPa, 3.9 MPa and 3.7 MPa, at shotcrete ages of 8 h, 24 h and 27 h, respectively. The total strain consists of the instantaneous strain due to load application, the autogenous shrinkage strain, the thermally induced strain due to hydration and the basic creep strain, as explained in [18].

**Figure 8:**
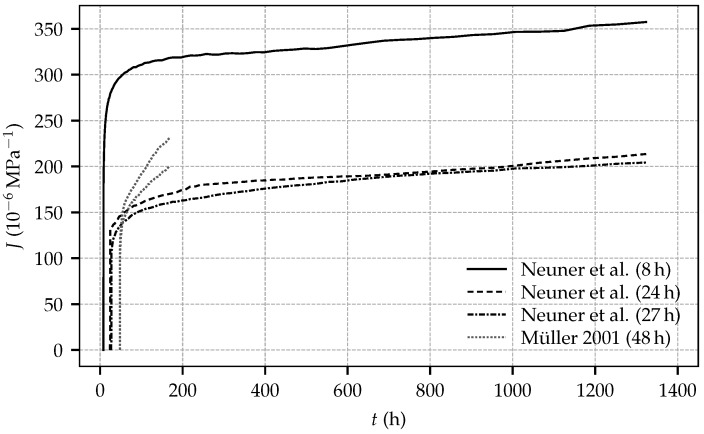
Compliance functions determined from the creep tests of the present experimental program, started at shotcrete ages of 8 h, 24 h and 27 h, and compliance functions from creep test series 4/2, conducted on two specimens by Müller, started at the shotcrete age of 48 h.

The curves representing the total strain determined on the loaded specimens are diverging slightly due to different magnitudes of the applied sustained stresses. From the total strain determined in a creep test, the compliance function, defined as load-induced time-dependent strain per unit stress [19], can be computed by subtracting the total strain determined in the respective shrinkage test and by dividing the so-obtained result by the stress applied in the creep test. Figure 8 shows the computed compliance functions for the three creep tests and, for comparison, the compliance functions obtained from the experimental data of the first load step of creep test 4/2 on two identical specimens by Müller [11], which were loaded at the shotcrete age of 48 h, keeping the load constant for 120 h. Only this first load step can be used for computing the compliance functions, since nonlinear creep effects due to high applied stresses occurred during further load steps. It can be seen that the computed compliance functions from the creep tests of the present experimental program exhibit the same slope after loading. Furthermore, the curves obtained from the creep tests, started at shotcrete ages of 24 h and 27 h, are nearly identical. By contrast, the compliance functions obtained from the creep tests by Müller are diverging and the respective curves in Figure 8 reveal the duration of the first load step to be too short for the assessment of the material compliance functions.

## 4. Constitutive Models for Shotcrete

Three advanced shotcrete models are considered in the present paper: (i) the model by Meschke [2], (ii) the model by Schädlich and Schweiger [3] and (iii) the SCDP (Shotcrete Damage Plasticity) model [4].

### 4.1. The Meschke Model

A three-dimensional constitutive model for shotcrete based on multisurface viscoplasticity theory was proposed by Meschke [2]. Validation based on experimental data from the literature and application of the model are reported in [20]. The model is capable of representing the evolution of stiffness and strength, shrinkage, creep, hardening material behavior in compressive and mixed loading and softening behavior in tensile loading. Associated flow rules are employed to describe viscoplastic flow. The evolution of material stiffness is based on an empirical law given as

(1) $$\begin{array}{cc} E(t) & =E^{\left(28\right)}\;\beta_{E}\left(t\right),\\ \beta_{E}\left(t\right) & =\{\begin{array}{cc} \beta_{E}^{I}=c_{E}t+d_{E}t^{2}, & \mathrm{if}\;t\leq t_{E},\\ \beta_{E}^{\mathrm{II}}={\left(a_{E}+\frac{b_{E}}{t-\Delta t_{E}}\right)}^{-0.5}, & \mathrm{if}\;t_{E}<t\leq 672\;h,\\ \beta_{E}^{\mathrm{III}}=1, & \mathrm{otherwise}, \end{array} \end{array}$$

in which *t* is the shotcrete age in hours, and $t_{E}$ and $\Delta t_{E}$ are model parameters for the early age behavior to describe an initially delayed start of the evolution of the Young’s modulus. Parameters $a_{E}$, $b_{E}$, $c_{E}$ and $d_{E}$ are model parameters to ensure a smooth transition at $t=t_{E}$:

(2) $$\begin{array}{cccc} a_{E} & =\frac{1-\frac{28-\Delta t_{E}/24}{1-\Delta t_{E}/24}{\left(\frac{E^{\left(1\right)}}{E^{\left(28\right)}}\right)}^{2}}{\left(1-\frac{28-\Delta t_{E}/24}{1-\Delta t_{E}/24}\right){\left(\frac{E^{\left(1\right)}}{E^{\left(28\right)}}\right)}^{2}}, & c_{E} & =\frac{2\beta_{E}^{\mathrm{II}}}{t_{E}}-\frac{d\beta_{E}^{\mathrm{II}}}{dt}{|}_{t=t_{E}},\\ b_{E} & =\left(672-\Delta t_{E}\right)\left(1-a_{E}\right), & d_{E} & =\frac{\frac{d\beta_{E}^{\mathrm{II}}}{dt}{|}_{t=t_{E}}\;t_{E}-\beta_{E}^{\mathrm{II}}}{t_{E}^{2}}. \end{array}$$

Therein, E^(1)^ and E^(28)^ denote the Young’s modulus at the age of one day and 28 days, respectively.

The evolution of the uniaxial compressive strength $f_{\mathrm{cu}}\left(t\right)$ is formulated for two time regimes: prior to the shotcrete age of 24 h, the recommendation reported in [15] is followed, whereas, after 24 h, the empirical approach by Oluokon et al. [21] is employed:

(3) $$f_{\mathrm{cu}}\left(t\right)=\{\begin{array}{cc} f_{\mathrm{cu}}^{\left(1\right)}{\left(\frac{t+0.12}{24}\right)}^{0.72453}, & \mathrm{if}\;t\leq 24\;h,\\ a_{c}\;\exp\left(-b_{c}/t\right), & \mathrm{otherwise}. \end{array}$$

Parameters $a_{c}$, $b_{c}$ are computed from the uniaxial compressive strength at the age of one day and 28 days, i.e., $f_{\mathrm{cu}}^{\left(1\right)}$ and $f_{\mathrm{cu}}^{\left(28\right)}$:

(4) $$\begin{array}{cccc} a_{c} & =\frac{f_{\mathrm{cu}}^{\left(28\right)}}{\exp\left(\ln\left(\frac{f_{\mathrm{cu}}^{\left(1\right)}}{f_{\mathrm{cu}}^{\left(28\right)}}\right)/27\right)}, & b_{c} & =-\left(672/27\right)\;\ln\left(\frac{f_{\mathrm{cu}}^{\left(1\right)}}{f_{\mathrm{cu}}^{\left(28\right)}}\right). \end{array}$$

Creep of shotcrete is modeled by a Duvaut–Lions type viscoplastic formulation, governed by the viscosity parameter η. Since in this model creep strains are viewed as temporally delayed plastic strains, it follows that creep due to sustained stresses within the elastic domain is neglected.

Shrinkage of shotcrete is described by means of the semi-empirical model proposed by Bažant and Panula [22], formulated on the basis of the three material parameters shrinkage half time $\tau_{\mathrm{shr}}$, ultimate shrinkage strain $\varepsilon_{\infty }^{\mathrm{shr}}$ and humidity dependent parameter $k_{h}$.

### 4.2. The Schädlich Model

Schädlich and Schweiger [3] proposed a viscoelastic-plastic material model for shotcrete, able to represent the evolution of material stiffness and strength, creep, shrinkage and plastic material behavior including hardening and softening, the latter formulated using non-associated plastic flow rules.

The evolution of material stiffness is modeled by a time-dependent Young’s modulus according to the CEB-FIP model code 1990 [23]:

(5) $$E\left(t\right)=E^{\left(28\right)}\;\exp\left(s_{\mathrm{stiff}}\left(1-\sqrt{28/t}\right)\right),$$

in which $E^{\left(28\right)}$ is the Young’s modulus at the age of 28 days, *t* represents the time in days and $s_{\mathrm{stiff}}$ is a model parameter computed as

(6) $$s_{\mathrm{stiff}}=\frac{-\ln(E^{\left(1\right)}/E^{\left(28\right)})}{\sqrt{28}-1},$$

with $E^{\left(1\right)}$ as the Young’s modulus at the age of one day.

The evolution of the uniaxial compressive strength $f_{\mathrm{cu}}\left(t\right)$ is described similarly to the evolution of the Young’s modulus by

(7) $$f_{\mathrm{cu}}\left(t\right)=f_{\mathrm{cu}}^{\left(28\right)}\;\exp\left(s_{\mathrm{strength}}\left(1-\sqrt{28/t}\right)\right),$$

in which $f_{\mathrm{cu}}^{\left(28\right)}$ is the uniaxial compressive strength at the age of 28 days and $s_{\mathrm{strength}}$ is a model parameter computed as

(8) $$s_{\mathrm{strength}}=\frac{-\ln(f_{\mathrm{cu}}^{\left(1\right)}/f_{\mathrm{cu}}^{\left(28\right)})}{\sqrt{28}-1},$$

with $f_{\mathrm{cu}}^{\left(1\right)}$ as the uniaxial compressive strength at the age of one day.

The evolution of material ductility is represented by a time-dependent reduction of the plastic strain at peak stress in uniaxial compression, $\varepsilon_{\mathrm{cpu}}^{p}$. It is formulated in terms of linear interpolation between the values of $\varepsilon_{\mathrm{cpu}}^{p}$ at the age of 1 h, 8 h and 24 h, i.e., $\varepsilon_{\mathrm{cpu}}^{p(1)}$, $\varepsilon_{\mathrm{cpu}}^{p(8)}$ and $\varepsilon_{\mathrm{cpu}}^{p(24)}$.

Shrinkage of shotcrete is modeled by the American Concrete Institute (ACI) committee 209 recommendation [19], based on the two material parameters ultimate shrinkage strain $\varepsilon_{\infty }^{\mathrm{shr}}$ and shrinkage half time $t_{50}^{\mathrm{shr}}$.

Nonlinear creep is described using a viscoelastic approach derived from the Eurocode 2 guideline [24]. It is formulated by means of the creep coefficient $\varphi^{\mathrm{cr}}$ and creep half time $t_{50}^{\mathrm{cr}}$.

### 4.3. The SCDP Model

The SCDP model is based on (i) the damage plasticity model for concrete by Grassl and Jirásek [25], (ii) the shrinkage model by Bažant and Panula [22] and (iii) a modified version of the solidification theory by Bažant and Prasannan [26,27]. Employing the combination of plasticity theory and isotropic continuum damage theory, non-associated plastic flow and isotropic hardening are modeled by the plasticity part of the model, and stiffness and strength degradation are described by means of the continuum damage part of the model.

The evolution of material stiffness and creep of shotcrete are described by a modified version of the solidification theory. The respective compliance function $J(t,t^{\prime })$, which relates the load-induced strain at time *t* (given in days) to a constant stress applied at time $t^{\prime }$, is expressed as

(9) $$J\left(t,t^{\prime }\right)=q_{1}+q_{2}\;Q\left(t,t^{\prime }\right)+q_{3}\;\ln\left(1+{\left(t-t^{\prime }\right)}^{0.1}\right)+q_{4}\;\ln\left(\frac{t}{t^{\prime }}\right).$$

The compliance function contains the four compliance parameters $q_{1}$, $q_{2}$, $q_{3}$ and $q_{4}$. Parameter $q_{1}$ is equal to the instantaneous, asymptotic material compliance, $q_{2}$ and $q_{3}$ are associated with short-term creep, and thus govern the evolution of material stiffness, and $q_{4}$ is related to long-term creep. For normal concrete, an estimation scheme for the compliance parameters based on the concrete composition is reported in [28] and extended in [29]. Although this estimation procedure was not developed for shotcrete, the obtained parameters may serve as a rough first estimate. Function $Q(t,t^{\prime })$ represents a binomial integral, which cannot be expressed in closed form, as discussed in [26]. However, it can be approximated for short time periods $t-t^{\prime }$ as $Q\left(t,t^{\prime }\right)\approx \tau {\left(t\right)}^{-0.5}\;\ln\left(1+{\left(t-t^{\prime }\right)}^{0.1}\right)$ (see [4]).

The solidification theory is modified by means of the time transformation function τ(t) in the SCDP model in order to take into account both an initial delay in the evolution of material stiffness and the subsequent faster hydration of shotcrete compared to normal concrete. Function τ(t) is defined as

(10) $$\tau \left(t\right)=\{\begin{array}{cc} \tau_{r}, & \mathrm{if}\;t\leq t_{r},\;\\ \frac{-2\tau_{p}+2\tau_{r}+\Delta t_{p}}{\Delta t_{p}^{3}}{\left(t-t_{r}\right)}^{3}+\frac{3\tau_{p}-3\tau_{r}-\Delta t_{p}}{\Delta t_{p}^{2}}{\left(t-t_{r}\right)}^{2}+\tau_{r}, & \mathrm{if}\;t_{r}<t\leq t_{p},\;\\ \frac{2(\tau_{p}-1)}{\Delta t_{a}^{3}}{\left(t-t_{p}\right)}^{3}-\frac{3(\tau_{p}-1)}{\Delta t_{a}^{2}}{\left(t-t_{p}\right)}^{2}+\left(t-t_{p}\right)+\tau_{p}, & \mathrm{if}\;t_{p}<t\leq t_{a},\;\\ t, & \mathrm{otherwise}, \end{array}$$

with $t_{p}=1\;d$, $t_{r}=0.1\;d$, $t_{a}=\max\;\left(28,\;3\;\tau_{p}-2\right)$, $\Delta t_{p}=t_{p}-t_{r}$, $\Delta t_{a}=t_{a}-t_{p}$, $\tau_{r}$ = 10^−2^ d and $\tau_{p}$ is given as

(11) $$\tau_{p}={\left(\frac{1/E^{\left(1\right)}-q_{1}}{q_{2}\ln\left(1+\Delta t^{0.1}\right)}-\frac{q_{3}}{q_{2}}\right)}^{-2}.$$

Therein, $E^{\left(1\right)}$ denotes the Young’s modulus at the age of 1 day, which can be considered as the fifth material parameter of the modified solidification theory, and Δt denotes a short time period for computing the effective stiffness: unlike for the Schädlich model and the Meschke model, the concept of a distinct time-dependent Young’s modulus is not applicable for the SCDP model. Rather, the Young’s modulus is approximated by the effective stiffness, computed for a short time period Δt from the compliance function J(t+Δt,t) of the modified solidification theory in Equation (9) as $E\left(t\right)\approx J{\left(t+\Delta t,t\right)}^{-1}$. Neglecting the effect of long-term creep on the effective stiffness over short time periods, departing from Equation (9), the Young’s modulus at time *t* can be approximated as

(12) $$E\left(t\right)\approx {\left(q_{1}+\ln\left(1+\Delta t^{0.1}\right)\;\left(q_{2}\;\tau {\left(t\right)}^{-0.5}+q_{3}\right)\right)}^{-1}.$$

Recommended values for Δt are given in the literature within the range of 10^−3^ d [28] to 10^−2^ d [29].

For describing the evolution of material strength, an empirical formulation is employed, which is derived from the relation used by Meschke [2] for describing the evolution of the Young’s modulus. It is formulated by means of the shotcrete age *t* (given in days) and the uniaxial compressive strength at the age of one day and 28 days, $f_{\mathrm{cu}}^{\left(1\right)}$ and $f_{\mathrm{cu}}^{\left(28\right)}$, respectively:

(13) $$\begin{array}{cc} f_{\mathrm{cu}}\left(t\right) & =f_{\mathrm{cu}}^{\left(28\right)}\;\beta_{f}\left(t\right),\\ \beta_{f}\left(t\right) & =\{\begin{array}{cc} \beta_{f}^{I}=r_{f}+c_{f}t+d_{f}t^{2}, & \mathrm{if}\;t\leq t_{f},\\ \beta_{f}^{\mathrm{II}}={\left(a_{f}+\frac{b_{f}}{t-\Delta t_{f}}\right)}^{-0.5}, & \mathrm{if}\;t_{f}<t\leq 28\;d,\\ \beta_{f}^{\mathrm{III}}=1, & \mathrm{otherwise}. \end{array} \end{array}$$

The model parameters are given as $r_{f}={\mathrm{10}}^{-2}$ and

(14) $$\begin{array}{cccc} a_{f} & =\frac{1-\frac{28-\Delta t_{f}}{1-\Delta t_{f}}{\left(\frac{f_{\mathrm{cu}}^{\left(1\right)}}{f_{\mathrm{cu}}^{\left(28\right)}}\right)}^{2}}{\left(1-\frac{28-\Delta t_{f}}{1-\Delta t_{f}}\right){\left(\frac{f_{\mathrm{cu}}^{\left(1\right)}}{f_{\mathrm{cu}}^{\left(28\right)}}\right)}^{2}}, & c_{f} & =\frac{d\beta_{f}^{\mathrm{II}}}{dt}{|}_{t=t_{f}}-\frac{2\beta_{f}^{\mathrm{II}}\left(t_{f}\right)}{t_{f}},\\ b_{f} & =\left(28-\Delta t_{f}\right)\left(1-a_{f}\right), & d_{f} & =\frac{\frac{d\beta_{f}^{\mathrm{II}}}{dt}{|}_{t=t_{f}}\;t_{f}-\beta_{f}^{\mathrm{II}}\left(t_{f}\right)+r_{f}}{t_{f}^{2}}. \end{array}$$

Therein, $f_{\mathrm{cu}}^{\left(1\right)}$ and $f_{\mathrm{cu}}^{\left(28\right)}$ denote the experimentally determined values for $f_{\mathrm{cu}}\left(t\right)$ at the age of one day and 28 days, respectively, and parameters $t_{f}$ and $\Delta t_{f}$ are proposed as $t_{f}$ = 0.25 d and $\Delta t_{f}$ = 0.18 d. Identical to the model by Schädlich and Schweiger [3], the evolution of material ductility is modeled by a temporal decrease of the plastic strain at peak stress in uniaxial compression, $\varepsilon_{\mathrm{cpu}}^{p}$.

Shrinkage is described by the semi-empirical model by Bažant and Panula [22], identical to the shotcrete model by Meschke.

An overview of the formulations governing the time-dependent material behavior in the three shotcrete models is provided in Table 3.

## 5. Comparison of Experimental and Computed Shotcrete Behavior

The identification of the material parameters for the shotcrete models closely follows the scheme presented in [4]. It is characterized by identifying parameters related to a material property exclusively from the respective experimental test on that property. In the following, all presented numerical results were obtained at material point level in incremental iterative simulations of the experimental tests. The constitutive models are integrated numerically by means of the fully implicit Euler scheme within the return mapping algorithm. The solidification theory employed by the SCDP model is implemented by means of a discrete Kelvin chain, as described in [30].

**Figure 9:**
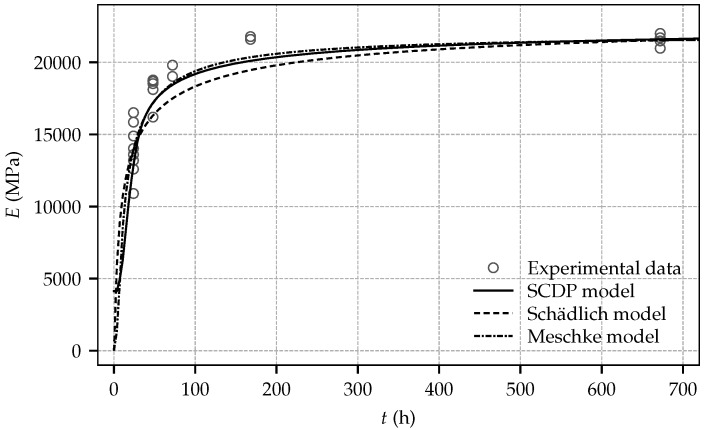
Evolution of the Young’s modulus: experimental results and computed numerical results based on the calibrated shotcrete models.

**Figure 10:**
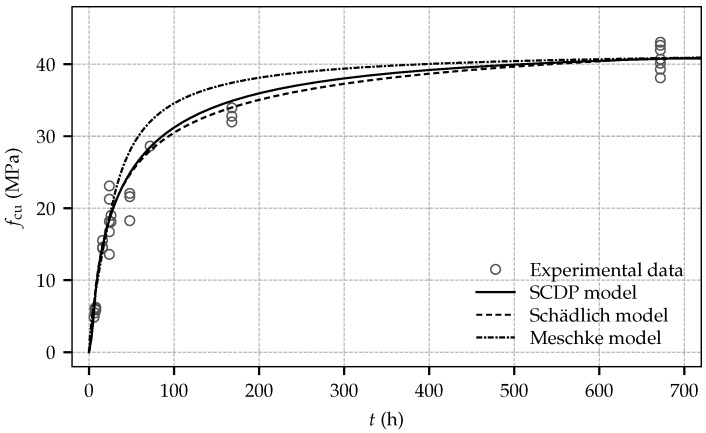
Evolution of the uniaxial compressive strength: experimental results and computed numerical results based on the calibrated shotcrete models.

For validation of the shotcrete models with respect to the measured evolution of stiffness and strength, the mean values of the Young’s modulus and the uniaxial compressive strength at the age of one day and 28 days, specified in Table 2, are employed. In addition, for the SCDP model, following the parameter estimation procedure by Bažant and Baweja, compliance parameter $q_{3}$ is computed as $q_{3}=3.03\times 10^{-6}$ MPa^−1^. By employing a least square optimization, parameters $q_{1}$ and $q_{2}$ are identified as $q_{1}=42.20\times 10^{-6}$ MPa^−1^ and $q_{2}=41.10\times 10^{-6}$ MPa^−1^. They assure a good agreement between the predicted and the observed evolution of the Young’s modulus between one day and 28 days. For comparison of the predicted evolution of the stiffness by the SCDP model, a time period of 10^−3^ d is chosen to determine the effective stiffness, in accordance with [28] and the approximate duration of load application during the experimental tests. Parameters $\Delta t_{E}$ and $t_{E}$ of the Meschke model are chosen as their default values proposed in [20] as $t_{E}$ = 8 h and $\Delta t_{E}$ = 6 h, since identification of them requires sufficient experimental data at a material age of a couple of hours. Figure 9 and Figure 10 show the predicted results for the evolution of stiffness and strength and for comparison the respective experimental data. While the SCDP model and the Meschke model predict very similar values of the Young’s modulus between shotcrete ages of one day and 28 days, the Schädlich model slightly underestimates the material stiffness in that time period. Furthermore, it can be seen that in contrast to the Meschke model and the Schädlich model, the SCDP model assumes a certain stiffness already at zero age, governed by model parameter $\tau_{r}$.

Very similar evolutions of the uniaxial compressive strength are obtained by the SCDP model and the Schädlich model, which are in good agreement with the experimental data. In contrast, the evolution predicted by the Meschke model is somewhat overestimating the actual material strength observed in the experiments.

**Figure 11:**
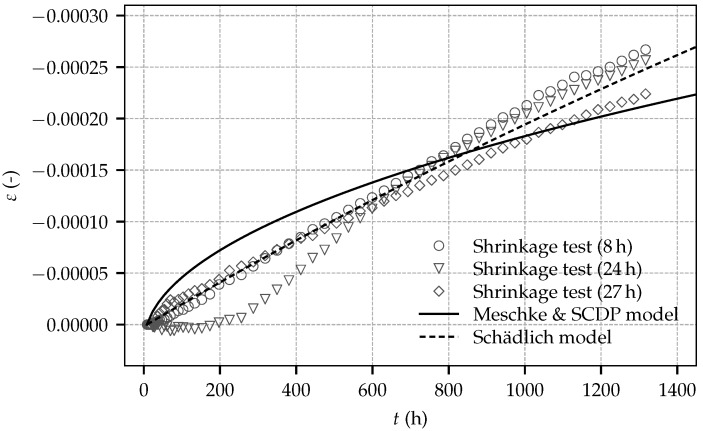
Evolution of the total strain determined on sealed, load-free specimens: experimental results and computed numerical results based on the calibrated shotcrete models.

A least square optimization is employed to identify the shrinkage parameters from the test data of the shrinkage tests started at shotcrete ages of 8 h and 27 h. The experimental data of the shrinkage test started at 24 h is neglected due to the initial delay of the evolution of the strain. Accordingly, parameters $\varepsilon_{\infty }^{\mathrm{shr}}$ and $t_{50}^{\mathrm{shr}}$ of the Schädlich model are obtained as $\varepsilon_{\infty }^{\mathrm{shr}}=-0.0019$ and $t_{50}^{\mathrm{shr}}$ = 8645 h. In contrast to the ACI model, which is employed by the Schädlich model, the Bažant–Panula model, employed by the SCDP model and the Meschke model, is not able to represent the low curvature of the experimental data: For this reason, for the SCDP model and the Meschke model, an ultimate shrinkage strain $\varepsilon_{\infty }^{\mathrm{shr}}$ is assumed as $\varepsilon_{\infty }^{\mathrm{shr}}=-0.002$ and the humidity dependent parameter $k_{h}$ is set to $k_{h}=1.0$. Thereby, a shrinkage half time of $\tau_{\mathrm{shr}}$ = 4082 h is identified. Experimental results of the shrinkage tests and the computed numerical results by the calibrated shotcrete models are shown in Figure 11. It can be seen that the Schädlich model is able to represent the evolution of the shrinkage strain better than the SCDP model and the Meschke model.

**Figure 12:**
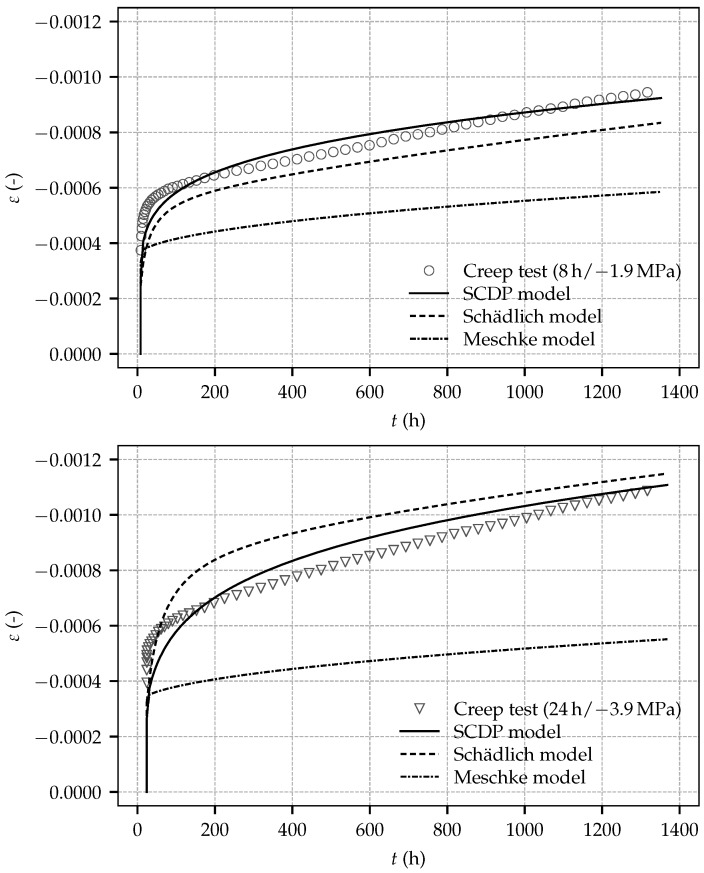

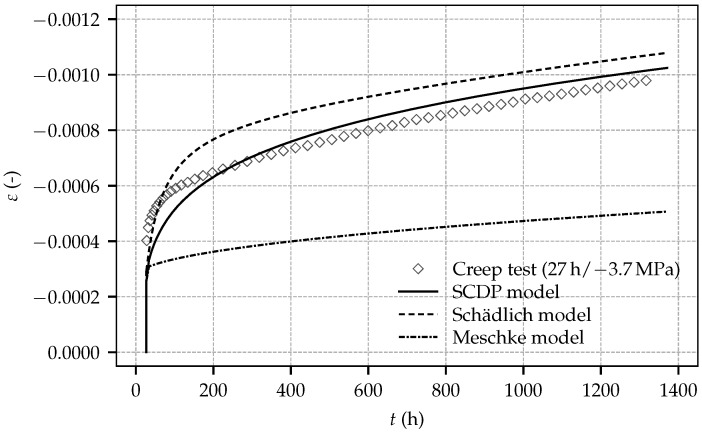
Evolution of the total strain determined on sealed, loaded specimens: Experimental results from the creep tests started at shotcrete ages of 8 h (**top**), 24 h (**middle**) and 27 h (**bottom**) and the respective computed numerical results based on the calibrated shotcrete models.

For calibration of the creep behavior, the creep parameters are identified from the test data of the three creep tests employing a least square optimization. However, the respective parameters are exclusively identified from the time-dependent strain after application of the load, i.e., the instantaneous strain due to load application is not considered in the identification procedure. The motivation for this approach becomes clear especially for the Meschke model and the Schädlich model: if the instantaneous strain was considered in the calibration procedure, an erroneous compensation of the discrepancy in the instantaneous strain between experimental results and predicted numerical results through overly high creep rates would be the consequence of the employed least square optimization. Due to the viscoplastic formulation of the Meschke model, the ratio of uniaxial compressive yield stress to uniaxial compressive strength, $f_{\mathrm{cy}}/f_{\mathrm{cu}}$, has a considerable influence on the predicted creep behavior. Similar to the identification procedure presented in [4] and in agreement with the proposed parameter range in [20], $f_{\mathrm{cy}}/f_{\mathrm{cu}}=0.1$ is assumed for all three shotcrete models. For the SCDP model and the Schädlich model, the material parameters governing the evolution of material ductility, $\varepsilon_{\mathrm{cpu}}^{p(1)}$, $\varepsilon_{\mathrm{cpu}}^{p(8)}$ and $\varepsilon_{\mathrm{cpu}}^{p(24)}$ are chosen as the default values proposed in [3]: $\varepsilon_{\mathrm{cpu}}^{p(1)}=-30.0\times 10^{-3}$, $\varepsilon_{\mathrm{cpu}}^{p(8)}=-0.7\times 10^{-3}$ and $\varepsilon_{\mathrm{cpu}}^{p(24)}=-0.7\times 10^{-3}$. For the SCDP model, the long-term creep compliance parameter $q_{4}$ is identified as $q_{4}=33.95\times 10^{-6}$ MPa^−1^. For the Schädlich model, the creep half time $t_{50}^{\mathrm{cr}}$ is assumed as the value proposed in [3] as $t_{50}^{\mathrm{cr}}$ = 36 h. Thereby, $\varphi^{\mathrm{cr}}$ is identified from the experimental data as $\varphi^{\mathrm{cr}}=2.62$. Remarkably, it is nearly identical to the one proposed as $\varphi^{\mathrm{cr}}=2.6$ in [3], calibrated on the basis of the creep tests by Aldrian [7]. For the Meschke model, the viscosity parameter η of the Meschke model is assumed as η = 0.25 h, in accordance with [20]. Figure 12 shows the experimental results together with the predicted numerical results by the shotcrete models.

The Schädlich model underestimates the total strain for the creep test started at 8 h, and overestimates the total strain for the creep tests started at 24 h and 27 h. For all creep tests, the SCDP underestimates the total strain immediately after loading and overestimates it thereafter. Long-term creep is represented by the Schädlich model and the SCDP model equally well, indicated by a similar slope of the predicted and measured long-term creep curves. Both the SCDP model and the Schädlich model are in good agreement with the experimental results, with the SCDP model slightly performing better.

In contrast, similarly as reported in [4] for the experimental tests by Müller [11], the viscoplastic formulation of the Meschke model is not able to represent the magnitude of the creep strain observed in the experiments. Creep, represented by viscoplastic flow, occurs only at the beginning of a creep test during a short time period after loading, and vanishes as soon as the uniaxial yield stress exceeds the applied stress due to the evolving material strength. Hence, identification of creep parameter η of the Meschke model is not possible on the basis of the present experimental data because of the low sustained stress levels in the present creep tests. As demonstrated in [20] on the basis of the creep tests by Huber [9] and in [5] by means of a finite element simulation of deep tunnel advance, the Meschke model represents creep of shotcrete very well for comparatively high prevailing stress levels.

## 6. Conclusions

A new experimental program on shotcrete and the evaluation of three advanced constitutive models for shotcrete, i.e., the Meschke model, the Schädlich model and the SCDP model, on the basis of the test data, were presented. The present paper closes the gap between the outdated experimental data on shotcrete available in the literature on the one hand and the nowadays available advanced constitutive models for representing the complex material behavior of shotcrete on the other hand. The new experimental program concentrated on the evolution of material properties of young shotcrete up to the age of 28 days as well as on the shrinkage and creep behavior, tested on sealed specimens up to the age of 56 days. Experiments were performed on two different types of specimens, i.e., drill cores sampled from spray boxes and directly sprayed specimens, the latter especially for experiments at very early shotcrete ages. The following conclusions can be drawn from the experimental program:

- The application of directly sprayed specimens from tubular molds proved to be a suitable alternative to drill cores from spray boxes. Furthermore, using sprayed specimens, experiments for determining the uniaxial compressive strength could be performed already six hours after casting.
- The tests on both the evolution of stiffness and strength are characterized by a remarkably small scatter of the determined material properties, especially for matured shotcrete.
- The observed evolution of the Young’s modulus indicates the ultimate stiffness to be fully developed at a material age of seven days.
- In contrast to material stiffness, the observed evolution of the uniaxial compressive strength shows a substantial increase of material strength between shotcrete ages of seven days and 28 days.
- Comparison of the experimental results of the present study to those presented by Müller, considering the latter as representative for shotcrete compositions used in the last decades, reveals the considerably improved performance of modern shotcrete compositions.

The presented calibration of three shotcrete models and the comparison of experimental and predicted results revealed the following insights:

- The determination of the material parameters for the three shotcrete models is characterized by identifying parameters related to a particular material property exclusively from the respective experimental test on that property. Hence, a simple least square approach can be employed for parameter identification from the standard lab tests presented in this paper. The only exception is the viscosity parameter η of the Meschke model, which must be calibrated on the basis of creep tests at higher stress levels, since creep due to stresses in the elastic domain is neglected by this model.
- All shotcrete models represent the evolution of material stiffness very well. The Young’s modulus is underestimated slightly by all models between shotcrete ages of one day and 28 days.
- Regarding the evolution of the uniaxial compressive strength, for all models, the computed response is in very good agreement with the experimental data. Both the SCDP model and the Schädlich model predict very similar results. A slight overestimation is obtained by the Meschke model between one day and 28 days.
- While the Schädlich model represents the shrinkage behavior very well, discrepancies between experimental results and the results predicted by the SCDP model and the Meschke model are striking.
- Both the Schädlich model and the SCDP model are able to represent the experimentally determined creep behavior very well. In contrast, the observed creep behavior cannot be reproduced using the viscoplastic Meschke model.

Although the presented experimental program covers a quite large variety of material properties of shotcrete, some material phenomena have received less attention so far, especially for nonlinear creep, which is considered as a very important subject in the context of tunneling. However, no profound experimental data is available in the literature. For this reason, a follow up experimental program, concentrating on that complex subject, is currently in preparation.

## Figures and Tables

**Table 1:** Composition of the shotcrete.

Property	Quantity	Unit
Water content	203	kg/m^3^
Cement content *CEM I 52.5N(sb)*	380	kg/m^3^
Limestone sand (0/4)	1031	kg/m^3^
Crushed limestone aggregates (4/8)	694	kg/m^3^
Additive *Fluasit*	40	kg/m^3^
Accelerator *Mapequick 043 FFG*	7.5–8.5	%

**Table 2:** Experimental program.

Tests for Determining the Evolution of	Number of Specimens	Mean Value (MPa)	Standard Deviation (SD) (MPa)
• shotcrete stiffness			
tests at the age of 1 day	8	13,943	1692
tests at the age of 2 days	5	18,054	954
tests at the age of 3 days	2	19,410	390
tests at the age of 7 days	2	21,485	295
tests at the age of 28 days	5	21,537	376
• shotcrete strength			
tests at the age of 6 h	3	5.46	0.51
tests at the age of 8 h	3	6.01	0.16
tests at the age of 16 h	3	14.85	0.48
tests at the age of 1 day	4	18.57	2.84
tests at the age of 2 days	3	20.64	1.69
tests at the age of 3 days	1	28.63	–
tests at the age of 7 days	3	32.90	0.80
tests at the age of 28 days	7	40.84	1.68
• shrinkage strains of sealed specimens			
started at the age of 8 h	1		
started at the age of 24 h	1		
started at the age of 27 h	1		
• creep strains of sealed specimens			
1.9 MPa applied at the age of 8 h	1		
3.9 MPa applied at the age of 24 h	1		
3.7 MPa applied at the age of 27 h	1		

**Table 3:** Summary and comparison of the shotcrete models.

Model	Evolution of Stiffness	Evolution of Strength	Shrinkage	Creep
Meschke	Empirical law	Guideline [15] and Oluokon et al. [21]	Bažant–Panula [22]	Viscoplastic formulation
Schädlich	CEB-FIP [23]	CEB-FIP [23]	ACI [19]	Eurocode 2 [24] based formulation
SCDP	Modified solidification theory [4,26]	Empirical law	Bažant–Panula [22]	Modified solidification theory [4,26]
